# Sarcopenia, sarcopenic obesity and the clinical outcome of the older inpatients with COVID-19 infection: a prospective observational study

**DOI:** 10.1186/s12877-024-05177-w

**Published:** 2024-07-04

**Authors:** Min Zong, Anda Zhao, Weijia Han, Yanqiu Chen, Tingwen Weng, Shijie Li, Lixin Tang, Jiang Wu

**Affiliations:** 1 Department of Clinical nutrition, Huadong Hospital affiliated to Fudan University, Shanghai, China; 2 Department of Geriatrics, Huadong Hospital affiliated to Fudan University, Shanghai, China

**Keywords:** Sarcopenia, Sarcopenic obesity, COVID-19 infection, Intubation, Mortality

## Abstract

**Objective:**

We aimed to investigate the impact of sarcopenia and sarcopenic obesity (SO) on the clinical outcome in older patients with COVID-19 infection and chronic disease.

**Methods:**

We prospectively collected data from patients admitted to Huadong Hospital for COVID-19 infection between November 1, 2022, and January 31, 2023. These patients were included from a previously established comprehensive geriatric assessment (CGA) cohort. We collected information on their pre-admission condition regarding sarcopenia, SO, and malnutrition, as well as their medical treatment. The primary endpoint was the incidence of intubation, while secondary endpoints included in-hospital mortality rates. We then utilized Kaplan-Meier (K-M) survival curves and the log-rank tests to compare the clinical outcomes related to intubation or death, assessing the impact of sarcopenia and SO on patient clinical outcomes.

**Results:**

A total of 113 patients (age 89.6 ± 7.0 years) were included in the study. Among them, 51 patients had sarcopenia and 39 had SO prior to hospitalization. Intubation was required for 6 patients without sarcopenia (9.7%) and for 18 sarcopenia patients (35.3%), with 16 of these being SO patients (41%). Mortality occurred in 2 patients without sarcopenia (3.3%) and in 13 sarcopenia patients (25.5%), of which 11 were SO patients (28%). Upon further analysis, patients with SO exhibited significantly elevated risks for both intubation (Hazard Ratio [HR] 7.43, 95% Confidence Interval [CI] 1.26–43.90, *P* < 0.001) and mortality (HR 6.54, 95% CI 1.09–39.38, *P* < 0.001) after adjusting for confounding factors.

**Conclusions:**

The prevalence of sarcopenia or SO was high among senior inpatients, and both conditions were found to have a significant negative impact on the clinical outcomes of COVID-19 infection. Therefore, it is essential to regularly assess and intervene in these conditions at the earliest stage possible.

**Supplementary Information:**

The online version contains supplementary material available at 10.1186/s12877-024-05177-w.

## Introduction

Sarcopenia, a degenerative condition commonly linked to the aging process, results in the deterioration of muscle mass, strength, and functionality. Its prevalence among the community-dwelling elderly varies with estimates ranging from roughly 6.6% to as high as 25.4% [1–5]. The incidence escalates in institutional settings such as nursing homes or among hospitalized older individuals, where it spans from 28 to 58% [6, 7]. Sarcopenia has been reported linked to longer hospital stays, diminished physical capabilities, a lower quality of life, and heightened long-term mortality across a broad spectrum of diseases, including COVID-19 [8–10]. Sarcopenic obesity (SO), a unique subtype of sarcopenia characterized by concurrent excess adiposity and diminished muscle mass or function, has garnered significant attention in the medical community. Since first described by Baumgartner in 2000 [11], the diagnostic criteria for SO have been refined in 2022 by the European Society for Clinical Nutrition and Metabolism (ESPEN) and the European Association for the Study of Obesity (EASO) [12]. Extensive multi-center cohort studies have demonstrated a correlation between SO and a heightened risk of various comorbidities, including cardiovascular disease, metabolic disorders, cognitive decline, arthritis, functional impairments, and pulmonary conditions [13]. Furthermore, SO has been linked to detrimental health outcomes in individuals with pre-existing cardiovascular and oncological conditions, such as a higher susceptibility to falls, fractures, and diminished self-care capabilities [14–16]. The implications of SO are profound, notably increasing the risk of all-cause mortality [13], with particularly pronounced effects observed in elderly patients within hospital settings [17] and in those with severe infections [18].

The geriatric, representing about 34% of all infections and 23% of deaths in COVID-19 infection, with those aged 70 and above being particularly vulnerable [19–22]. Literatures [23–25] have consistently demonstrated that age and sarcopenia significantly contribute to the risk of adverse outcomes in COVID-19 patients. These outcomes included extended hospital stays, increased likelihood of intensive care unit (ICU) admission, the requirement for intermittent mandatory ventilation (IMV), and higher mortality rates. To date, only one study has reported that SO might elevate the risk of cardiovascular disease and mortality among older patients with COVID-19 [26].

Despite growing interest, the prevalence and influence of SO on disease prognosis remain contentious, primarily due to the variability in diagnostic criteria [13]. The scarcity of domestic research on the prognostic implications of SO, particularly in relation to pulmonary diseases such as COVID-19 infection in the geriatric, highlights a significant gap in the literature. In response to the situation, we conducted a prospective observational cohort study. Our primary objective was to investigate the independent effects of sarcopenia or SO on the adverse outcomes of older patients hospitalized with pulmonary infections. This study aimed to provide empirical evidence that could inform early, routine screening, evaluation, and intervention strategies for sarcopenia or SO in geriatric population.

## Materials and methods

### Study design and participants

From November 1, 2022, to January 31, 2023, we conducted a prospective observational study on the older patients who were consecutively admitted to Huadong Hospital due to acute COVID-19 infections. These patients were part of a previously established comprehensive geriatric assessment (CGA) cohort.

To be eligible for inclusion in the study, participants had to meet the following criteria: (1) age 65 years or older, (2) confirmed diagnosis of coronavirus-2 (SARS-CoV-2) infection, determined using polymerase chain reaction (PCR), (3) had CGA records within 6 months before admission. Patients who met any of the following criteria were excluded from the study: (1) diagnosed with end-stage renal disease(creatinine clearance rate(CCr) < 30 ml/min), moderate to severe liver failure (Child-Pugh B or C), and malignant tumors, (2) underwent surgery and radiotherapy within the last three months, (3) missing data on the assessment of sarcopenia, frailty, and malnutrition.

The study adhered to the principles and guidelines outlined in the Declaration of Helsinki and its subsequent amendments, and the protocol was approved by the Ethics Committee of Huadong Hospital (2023K199). Our methodology strictly followed the STROBE (Strengthening the Reporting of Observational Studies in Epidemiology) guidelines to ensure the transparency and rigor of our observational research.

### Calculation of the sample size

We utilized PASS 15.0 software to calculate the sample size, employing the “Tests for Two Survival Curves Using Cox’s Proportional Hazards Model” function. Based on a prior study [27], we hypothesized a hazard ratio for intubation of 2.867, with an assumed progression to intubation of 22% among patients without sarcopenia and 68% among those with sarcopenia. The calculated minimum sample size was 80 patients to ensure adequate statistical power (α = 0.05, β = 0.1). Considering a potential dropout rate of 20%, the initial sample size was adjusted to a minimum of 107 participants.

### Data collection

#### Demographic and clinical data

We obtained data on the participant’s age, sex, medical history, body mass index (BMI), as well as comorbidities such as hypertension (HBP), hyperlipidemia (HLP), coronary artery disease (CAD), chronic obstructive pulmonary disease (COPD), and chronic kidney disease (CKD). Blood test results within 48 h after hospitalization were recorded, which include virus PCR test, white blood cell count, lymphocyte count percentage, fibrinogen levels, and D-dimer level. Low lymphocyte count percentage (percentage of lymphocyte count less than 20%), hyperfibrinogenemia (fibrinogen levels above 4 g/L), and hyperdimeremia (D-dimer levels above 0.55 mg/L) were indicative of abnormalities. During the hospitalization, we documented the medical procedures and their results, such as the administration of immunoglobulins, glucocorticoids, antimicrobials, and Paxlovid. Additionally, we closely monitored intubation and assessed the in-hospital mortality rate. This comprehensive record-keeping ensures a thorough evaluation of the treatment and its outcomes.

#### CGA records on sarcopenia, SO, malnutrition, and frailty

CGA records within six-month prior to admission were reviewed. Patients’ height, weight were measured using Height and Weight Scale (BSM370, Inbody Inc. Korea). BMI was calculated by weight (kg) / height (m^2^). Body composition indices such as appendicular skeletal muscle mass (ASM) and percent body fat (PBF) were evaluated using Multi-Frequency Bioelectrical Impedance Measurement (DSM-BIA) method (Inbody S10, Inbody Inc. Korea) [28]. Appendicular skeletal muscle mass index (ASMI) was calculated by ASM (kg) / height (m^2^). Grip hand strength (HGS) was assessed using a grip dynamometer (Jamar^®^ Hand Evaluation, USA) [29]. Based on the Asian Working Group for Sarcopenia (AWGS) [30], ASMI less than 7.0 kg/m^2^ (male) or 5.7 kg/m^2^ (female) was defined as decreased muscle mass. Diminished muscle strength is defined by a HGS measurement of less than 28 kg for males or 18 kg for females. Additionally, a 6-meter gait speed of less than 1.0 m/s was considered indicative of reduced muscular endurance. Sarcopenia was diagnosed through a reduction in either ASMI and HGS or ASMI and 6-meter gait speed. In accordance with the consensus statement by ESPEN & EASO [12], PBF exceeding 25% (male) or 35% (female) was considered obese status. SO was confirmed by a diagnosis of sarcopenia with obese status.

We employed the Global Leadership Initiative on Malnutrition (GLIM) framework [31] to assess the nutrition status of patients within the CGA cohort. The GLIM assessment incorporated three phenotypic factors: BMI, ASMI, and unintentional weight loss (within 6 months). For the etiologic type, the assessment considered dietary intake, gastrointestinal function, and the presence of elevated C-reactive protein (CRP > 10 mg/L). The presence and severity of malnutrition was diagnosed according to the established GLIM criteria.

Frailty was evaluated based on the five aspects of the Frail Scale [32, 33]: fatigue, resistance, ambulation, illnesses, and weight loss. A score of 3–5 indicates frailty, while a score of 1–2 suggests a pre-frail status.

### Statistical analysis

Initially, we employed the Kolmogorov-Smirnov test to assess the normality of the data distribution. Normally distributed data were described by mean and standard deviation, and the independent t-test was used to compare the two groups. Non-normally distributed data were expressed by median and interquartile range (IQR) (25-75th percentile), and Wilcoxon rank-sum test was used for the comparison of two independent groups. Categorical count data were reported as absolute frequencies and percentages. Chi-square tests were performed to compare categorical variables between two groups.

To evaluate the effects of independent variables on sarcopenia or SO, we utilized multivariate forward stepwise logistic regression models to estimate odds ratios (ORs) with their corresponding 95% confidence intervals (CIs). The selection of independent variables—such as age, sex, chronic conditions including HBP, CAD, CKD, and COPD, as well as geriatric syndromes like frailty and malnutrition—were selected based on previous literature [2–5]. Frailty was categorized as a binary variable, identified by a Frail scale score exceeding 3, while the remaining variables were treated as continuous.

Additionally, Cox proportional hazards regression analysis was applied to determine hazard ratios (HRs) along with their 95% CIs. For the estimation of intubation and mortality rates, Kaplan-Meier (K-M) survival curves were constructed, and any statistically significant differences between groups were assessed using the log-rank test. Two-sided *P*-values < 0.05 were considered statistically significant. The K-M curves were plotted using R Foundation for Statistical Computing (Version 4.2.0, Vienna, Austria), while other statistical analyses were executed with the SPSS software package (Version 22, IBM, Chicago, USA).

## Results

**Fig. 1:**
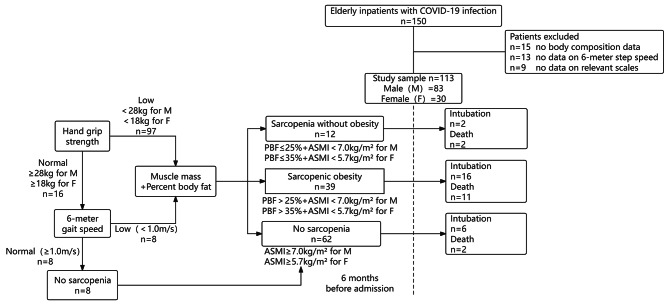
Flowchart describing participant recruitment This flowchart illustrates the participant enrollment methodology for our study. We enrolled a cohort of 113 elderly patients hospitalized with COVID-19, all of whom underwent a comprehensive geriatric assessment prior to admission, specifically six months before. Utilizing the pre-admission evaluations for sarcopenia, participants were categorized into three groups: non-sarcopenia group, sarcopenia group, and sarcopenic obesity group

The study encompassed a total of 113 patients who fulfilled the eligibility criteria, as depicted in Fig. 1. These patients had an average age of 89.6 ± 7.0 years, consisting of 83 males and 30 females. As shown in Tables 1 and 51 (45.1%) of the older were diagnosed with sarcopenia while 39 (34.5%) as having SO. According to the CGA record, 18 participants (15.9%) were found to be malnourished, all of whom were among those with sarcopenia. There were 5 cases of patients with moderate malnutrition and 3 cases with severe malnutrition. Additionally, 64 individuals (57.5%) were identified as frail, as determined by the Frail scale score.

**Table 1:** Characteristics of baseline according to sarcopenia and sarcopenic obesity

Parameters	Total (*n* = 113)	Non-sarcopenia (*n* = 62)	Sarcopenia (*n* = 51)	Sarcopenic obesity (*n* = 39)	t/χ² (c/d)	*P* (c/d)
General						
Age, y^a^	89.6 ± 6.9	87.1 ± 6.9	92.7 ± 5.7	93.2 ± 5.1	-4.67/-4.74	< 0.001/<0.001
Sex (M/F), n^b^	83/30	44/18	39/12	34/5	0.44/3.58	0.510/0.059
Comorbidities						
HBP, n(%)^b^	88 (77.9)	52 (83.9)	36 (70.6)	27 (69.2)	2.87/3.01	0.091/0.083
HLP, n(%)^b^	34 (30.1)	23 (37)	11 (21.6)	9 (23.1)	3.21/2.17	0.073/0.114
CAD, n(%)^b^	72 (63.7)	39 (62.9)	33 (64.7)	26 (66.7)	0.04/0.15	0.843/0.701
COPD, n(%)^b^	34 (30.1)	13 (20.9)	21 (41.2)	19 (48.7)	5.43/8.52	0.020/0.004
CKD, n(%)^b^	15 (13.3)	9 (14.5)	6 (11.8)	5 (12.8)	0.18/0.06	0.668/0.810
More than five co-morbidities, n(%)^b^	73 (75)	46 (79)	27 (69)	22 (71)	3.18/0.99	0.075/0.319
Related indicators						
BMI (kg/m^2^)^a^	23.4 ± 3.0	24.4 ± 2.6	22.2 ± 3.1	23.3 ± 2.5	4.16/2.15	< 0.001/0.034
CC (cm)^a^	31.6 ± 3.4	33.1 ± 2.6	29.7 ± 3.3	30.5 ± 2.5	5.88/4.70	< 0.001/<0.001
PBF (%)^a^	31.9 ± 9.5	29.9 ± 9.3	34.2 ± 9.2	37.3 ± 6.9	-2.46/-4.31	0.016/<0.001
Increased PBF, n(%)^b^	75 (66)	36 (58)	39 (76)	39 (100)	4.29/22.03	0.039/<0.001
ASMI (kg/m^2^)^a^	6.67 ± 1.30	7.41 ± 1.01	5.77 ± 1.02	5.92 ± 0.86	8.54/7.62	< 0.001/<0.001
Decreased ASMI, n(%)^b^	51 (51)	0 (0)	51 (100)	39 (100)	113.00/101.00	< 0.001/<0.001
Hand grip strength(kg)^a^	17.3 ± 9.7	20.7 ± 9.3	12.8 ± 8.4	11.6 ± 6.2	4.43/5.14	< 0.001/<0.001
Decreased grip strength, n(%)^b^	97 (85.8)	48 (77.4)	49 (96.1)	39 (100)	8.02/10.22	0.005/0.001
Malnutrition by GLIM, n(%)^b^	18 (15.9)	0 (0)	18 (35.3)	16 (41.0)	26.03/30.22	< 0.001/<0.001
Frailty, n(%)^b^	64 (57.5)	25 (40.3)	40 (78.4)	32 (82.1)	18.70/15.47	< 0.001/<0.001

HBP: hypertension; HLP: hyperlipidemia; CAD: coronary artery disease; COPD: chronic obstructive pulmonary disease; CKD: chronic kidney disease; BMI: body mass index; PBF: percent body fat; ASMI: appendicular skeletal muscle index; CC: calf circumference. A: continuous variables were presented as mean ± standard deviation: two independent sample t test were used; b: categorical variables were presented by number (%): pearson chi-square test for categorical data was used; c: Sarcopenia group compared to non-sarcopenia group; d: sarcopenic obesity group compared to non-sarcopenia group

Compared with the non-sarcopenia group, both the sarcopenia and SO groups had older age, higher prevalence of COPD, higher levels of PBF (*P* < 0.05). Notably, it was worth noting that both the sarcopenia and SO groups exhibited relatively higher rates of malnutrition (35% and 41%, respectively) and frailty (78% and 84%, respectively) (Table 1).

### Factors associated with sarcopenia and SO

First, in the univariate analysis (Table 1), age, COPD, malnutrition as defined by the GLIM criteria, and frailty were identified as independent variables that differentiated between the sarcopenia and non-sarcopenia groups. Sex was found to be differed between the SO and non-sarcopenia groups. These variables were subsequently subjected to multivariate logistic regression analysis.

The results showed that age (OR 1.19, 95%CI 1.07–1.33, *P* = 0.001) and frailty (OR 3.43, 95%CI 1.02–11.53, *P* = 0.046) were found to be associated with sarcopenia. On the other hand, age (OR 1.25, 95%CI 1.10–1.42, *P* = 0.001), male (OR 7.96, 95%CI 1.61–39.47, *P* = 0.011), and malnutrition by GLIM (OR 20.2, 95%CI 3.14–130.2, *P* = 0.002) were identified as significant factors related to SO, as shown in Table 2. Additional univariate analysis on the single variable revealed that glucocorticoid use demonstrated a significant correlation with two critical clinical outcomes: intubation and mortality (*P* < 0.01).

**Table 2:** Assocociated factors for sarcopenia and sarcopenic obesity

	Univariate associations with sarcopenia		Multivariate associations with sarcopenia		Univariate associations with SO		Multivariate associations with SO	
	OR (95% CI)	*P*	OR (95% CI)	*P*	OR (95% CI)	*P*	OR (95% CI)	*P*
Age	1.15(1.07–1.23)	0.000	1.19(1.07–1.33)	0.001	1.14(1.06–1.23)	< 0.001	1.25(1.10–1.42)	0.001
Sex (male)	1.33(0.57–3.11)	0.51			3.46(1.21–9.97)	0.02	7.96(1.61–39.5)	0.010
HBP	2.17(0.88–5.36)	0.09			0.48(0.19–1.19)	0.110		
HLP	0.47(0.20–1.08)	0.07			0.59(0.24–1.43)	0.24		
CAD	1.08(0.50–2.34)	0.84			1.22(0.54–2.75)	0.64		
COPD	2.64(1.15–6.04)	0.02			3.74(1.60–8.71)	0.002		
CKD	0.79(0.26–2.38)	0.67			0.94(0.29–2.98)	0.92		
Malnutrition by GLIM	/	/			25.0(5.35–117.2)	< 0.001	20.22(3.14–130.2)	0.002
Frailty	7.31(2.81–19.1)	0.00	3.43(1.02–11.5)	0.046	5.33(1.97–14.5)	0.001		

HBP: hypertension; HLP: hyperlipidemia; CAD: coronary artery disease; COPD: chronic obstructive pulmonary disease; CKD: chronic kidney disease;

/: unable to calculate; SO: sarcopenic obesity; OR: odds ratios; CI: confidence interval

### Impact of sarcopenia and SO on intubation and mortality

**Fig. 2:**
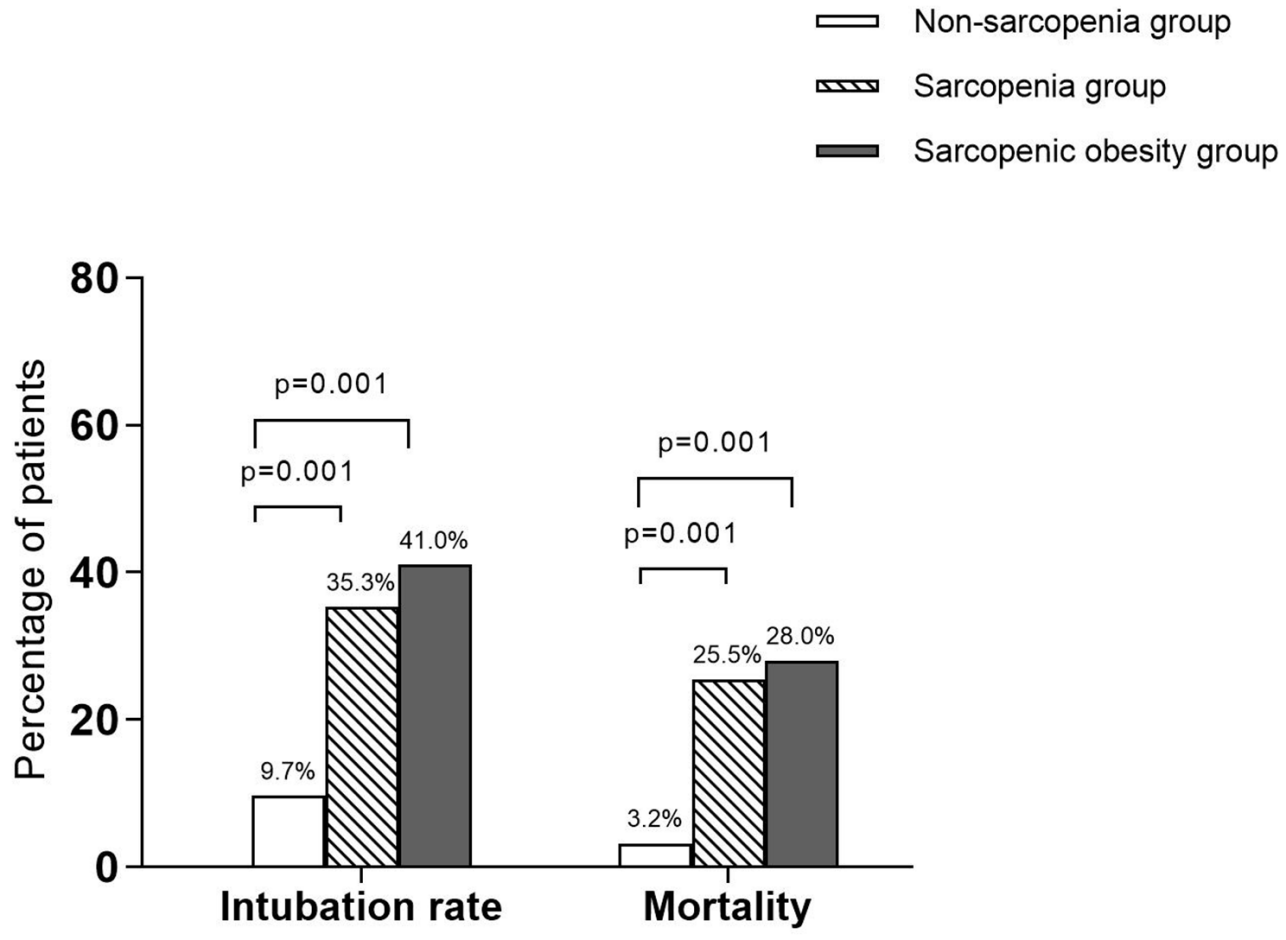
Intubation rate and mortality among three groups This bar graph illustrates the rate of intubation and mortality among three groups of elderly COVID-19 patients: those with no sarcopenia, those with sarcopenia, and those with sarcopenic obesity. Compared to the non-sarcopenia group, the rate of intubation and mortality were both significantly higher in the sarcopenia group, and the sarcopenic obesity group (*P* = 0.001)

**Fig. 3:**
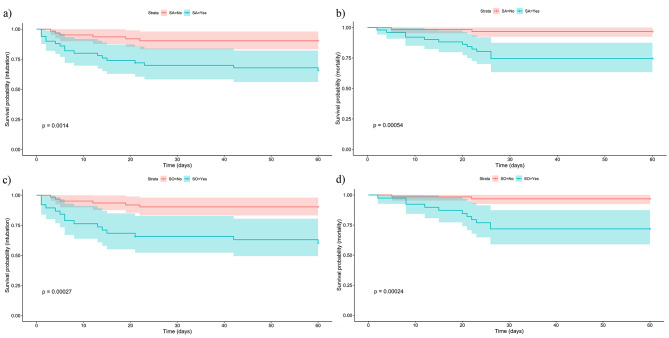
Kaplan-Meier survival estimates for intubation and mortality rates in patients (**a**) Intubation rates as estimated by Kaplan-Meier for patients with sarcopenia versus those without. (**b**) Mortality rates as estimated by Kaplan-Meier for patients with sarcopenia versus those without. (**c**) Mortality rates as estimated by Kaplan-Meier for patients with SO versus those without SO. (**d**) Comparative mortality rates as estimated by Kaplan-Meier between patients with and without SO

Throughout the observation period, 24 patients (21.2%) necessitated intubation, and 15 (13.2%) succumbed to their illness. Among all, patients presenting with sarcopenia and SO exhibited a greater propensity for intubation, with 18 and 16 cases respectively, and these conditions were associated with increased mortality rates, accounting for 13 and 11 respectively (Table 3; Fig. 2). Following adjustments for age, sex, BMI, malnutrition, and glucocorticoid use, Cox regression analyses further illustrated that sarcopenia was an independent predictor of intubation (HR 3.26, 95%CI 1.03–10.34, *P* < 0.001) and mortality (HR 4.03, 95%CI 1.21–13.40, *P* < 0.001). As a subtype of sarcopenia, SO emerged as a significantly stronger risk factor for both intubation (HR 7.43, 95%CI 1.26–43.90, *P* < 0.001) and death (HR 6.54, 95%CI 1.09–39.38, *P* < 0.001) (Table 4). K-M survival curves distinctly revealed that patients with either sarcopenia or SO, when compared to the non-sarcopenia group, faced a higher likelihood of intubation and a reduced likelihood of survival (*P* < 0.05) (Fig. 3).

**Table 3:** Characteristics after COVID-19 infection according to sarcopenia and sarcopenic obesity

Parameters of COVID-19	Total (*n* = 113)	Non-sarcopenia (*n* = 62)	Sarcopenia (*n* = 51)	SO (*n* = 39)	t/χ² (c/d)	*P* (c/d)
**Outcome**						
Intubation, n(%)^b^	24 (21.2)	6 (9.7)	18 (35.3)	16 (41.0)	10.98/13.81	0.001/0.001
Death, n(%)^b^	15 (13.3)	2 (3.2)	13 (25.5)	11 (28.0)	12.05/13.32	0.001/0.001
**Treatment**						
Immunoglobulin, n(%)^b^	54 (47.8)	26 (41.9)	28 (54.9)	23 (58.9)	1.89/2.78	0.170/0.095
Glucocorticoids, n(%) ^b^	29 (25.7)	11 (17.7)	18 (35.3)	16 (41)	4.52/6.63	0.034/0.010
Paxlovid, n(%)^b^	61 (53.9)	29 (46.7)	32 (62.7)	23 (58.9)	2.87/1.43	0.090/0.232
**Laboratory indicators**						
White blood cell count (10^9^/L)^a^	6.3 ± 2.3	5.8 ± 1.7	6.8 ± 2.8	6.9 ± 2.9	-2.04/-2.06	0.044/0.045
Low lymphocyte count percentage, n(%)^b^	37 (33)	16 (26)	21 (41)	19 (49)	3.00/5.55	0.083/0.018
Hyperfibrinogenemia, n(%)^b^	27 (25)	9 (15)	18 (35)	15 (38)	5.93/6.84	0.015/0.009
Hyperdimerinemia, n(%)^b^	69 (63)	30 (51)	39 (76)	29 (74)	7.68/5.42	0.020/0.047

SO: sarcopenic obesity. a: continuous variables were presented as mean standard deviation: two independent sample t test were used; b: categorical variables were presented by number (%): pearson chi-square test for categorical data was used; c: sarcopenia group compared to non-sarcopenia group; d: sarcopenic obesity group compared to non-sarcopenia group

**Table 4:** Associations between sarcopenia or sarcopenic obesity and clinical outcomes

	Non-sarcopenia (*n* = 62)	Sarcopenia (*n* = 51)	Sarcopenia vs. Non-sarcopenia			Sarcopenic Obesity (*n* = 39)	Sarcopenic Obesity vs. Non-sarcopenia		
			Unadjusted	Model 1	Model 2		Unadjusted	Model 1	Model 2
Intubation Events	6	18				16			
HR			4.07	4.01	3.26		8.82	9.63	7.43
95% CI			1.60, 10.34	1.26,12.81	1.03,10.34		1.99,39.12	1.62,57.19	1.26,43.90
Death Events	2	13				11			
HR			4.91	5.80	4.03		9.88	12.08	6.54
95% CI			1.90,12.67	1.80,18.71	1.21,13.40		2.19,44.62	2.06,70.75	1.09,39.38

Model 1 was adjusted for age, sex, body mass index, malnutrition by GLIM

Model 2 was adjusted for model 1 plus glucocorticoids use

## Discussion

Our study revealed a remarkably high prevalence of sarcopenia and SO among older patients hospitalized with COVID-19, and the presence of sarcopenia, especially SO independently influenced adverse outcomes of COVID-19, including intubation or death.

45% of the participants had sarcopenia, which was consistent with previous findings by Gingrich [34]. He found that about 42% of 100 hospitalized patients with gastrointestinal diseases and malignant tumors suffered from sarcopenia. But due to the plethora of previous diagnostic techniques used to assess obesity, such as BMI, PBF, and visceral fat area, the occurrence of obesity in healthy populations showed significant variations [35]. Recognizing the limited accuracy of BMI, ESPEN and EASO established PBF as the standardized method for determining SO in 2022 [12]. In our cohort study, it was observed that despite patients having a BMI within the normal range, the utilization of PBF revealed a prevalence of obesity of 34.5%, which was comparable to the 34.1% prevalence observed in patients receiving chemotherapy for esophageal cancer [36]. Li [37] documented that aging, a sedentary lifestyle, an unhealthy diet, and insulin resistance were identified as risk factors for obesity. The development of SO involved a complex mechanism. With aging, adipose tissue in the body tends to accumulate ectopically and redistribute in the liver and muscle. The presence of ectopic adipocytes not only disrupted the alignment of muscle fibers but also led to the release of adipokines, which in turn triggered a heightened pro-inflammatory response. This chronic inflammation ultimately caused dysfunction and apoptosis of myocytes. Furthermore, these cytokines could worsen the atrophy of adipose tissue, leading to a vicious cycle of localized hyperlipidemia and inflammation [38].

In our study, we found that both sarcopenia and SO were strongly associated with malnutrition and frailty, and independently contributed to an increased risk of intubation or death. This finding highlights the crucial role these two factors play in determining patient outcomes. Previous studies [8–10, 39] on sarcopenia had documented not only its negative impact on clinical outcomes, but also the coexistence of sarcopenia with other geriatric syndromes had been shown to further exacerbate the risk of disease severity and death. These relationships often involve reciprocal causation, where the syndromes coexist and worsen each other’s effects. When two or more co-morbidities, such as frailty, sarcopenia, and malnutrition, were present, the mortality rate increased to nearly 75% [40]. These findings highlight the vulnerability of older individuals with multiple geriatric syndromes to severe illness and death during the COVID-19 pandemic.

So far, the impact of SO on clinical outcomes remained unclear. Some studies [14, 41, 42] found a strong association between SO and an increased risk of osteoporosis, falls, fractures, and other related conditions, while others did not [43]. As to the pulmonary disease, a Korean cohort study [44] has identified SO as a determinant affecting pulmonary function, with a significant association to the development of restrictive pulmonary disease (OR 2.81, 95% CI 1.72–4.59). A multi-center retrospective cohort study [45] in the United States recognized SO as a predictive risk factor for extended hospital stays and an increased incidence of postoperative complications following lobectomy for lung cancer. Regarding pulmonary infection, Mayoral er al [26, 46] reported that SO, as opposed to obesity alone, was found to have a higher likelihood of increasing the expression of inflammatory cytokines, and might be associated with a poor prognosis in COVID-19 patients. This heightened inflammatory response in SO might contribute to exacerbating the immune and metabolic stress caused by COVID-19 which partially explain why patients with SO had higher risks than those with sarcopenia alone in our study. Given the aforementioned information, it is imperative to prioritize early CGA evaluations that identify conditions such as sarcopenia—particularly SO—as well as malnutrition and frailty. Timely intervention is essential for enhancing the quality of life and averting poor prognoses in geriatric patients with pulmonary infections. Additionally, it is also essential to vigorously pursue research into intervention strategies aimed at combating sarcopenia and SO, such as measures to alleviate inflammation status and improve body composition by optimizing lifestyle factors [47, 48].

Furthermore, This study bears some limitations to be acknowledged. Firstly, there may have been selection bias as the participants were recruited from a single center, which may not be representative of the larger population. Additionally, the sample size was relatively small, which may affect the statistical power and precision of the study. Secondly, the study design itself might have inherent limitations. For example, the data collection methods used may introduce measurement bias or confounding variables that were not accounted for. Lastly, the prediction formulas used in the study might need further refinement to improve their accuracy and reliability.

## Conclusions

The findings of our research underscore the prevalent incidence of sarcopenia and sarcopenic obesity among the geriatric population suffering from pulmonary diseases. It is noteworthy that both conditions have been established as independent predictors of adverse outcomes, including the necessity for intubation and the risk of mortality. Our study provides solid theoretical evidence advocating for the early adoption of routine screening, thorough assessment, and specific interventions for sarcopenia or SO in senior patients, extending to, but not limited to, those afflicted with pulmonary infections.

### Electronic supplementary material

Below is the link to the electronic supplementary material.

Supplementary Material 1

## Data Availability

The datasets used and/or analysed during the current study available from the corresponding author on reasonable request.
